# A service evaluation of transport destination and outcome of patients with post-ROSC STEMI in an English ambulance service

**DOI:** 10.29045/14784726.2020.06.5.1.32

**Published:** 2020-06-01

**Authors:** Anthony Platt

**Affiliations:** Yorkshire Ambulance Service NHS Trust

**Keywords:** out-of-hospital cardiac arrest, post-ROSC care, pPCI

## Abstract

**Background:**

In the UK, there are approximately 60,000 cases of out-of-hospital cardiac arrest (OHCA) each year. There is mounting evidence that post-resuscitation care should include early angiography and primary percutaneous coronary intervention (pPCI) in cases of OHCA where a cardiac cause is suspected. Yorkshire Ambulance Service (YAS) staff can transport patients with a return of spontaneous circulation (ROSC) directly to a pPCI unit if their post-ROSC ECG shows evidence of ST elevation myocardial infarction (STEMI). This service evaluation aimed to determine the factors that affect the transport destination, hospital characteristics and 30-day survival rates of post-ROSC patients with presumed cardiac aetiology.

**Methods:**

All patient care records (PCRs) previously identified for the AIRWAYS-2 trial between January and July 2017 were reviewed. Patients were eligible for inclusion if they were an adult non-traumatic OHCA, achieved ROSC on scene and were treated and transported by (YAS). Descriptive statistics were used to analyse the data.

**Results:**

478 patients met the inclusion criteria. 361/478 (75.6%) patients had a post-ROSC ECG recorded, with 149/361 (41.3%) documented cases of STEMI and 88/149 (59.1%) referred to a pPCI unit by the attending clinicians. 40/88 (45.5%) of referrals made were accepted by the pPCI units. Patients taken directly to pPCI were most likely to survive to 30 days (25/39, 53.8%), compared to patients taken to an emergency department (ED) at a pPCI-capable hospital (34/126, 27.0%), or an ED at a non-pPCI-capable hospital (50/310, 16.1%).

**Conclusion:**

Staff should be encouraged to record a 12-lead ECG on all post-ROSC patients, and make a referral to the regional pPCI-capable centre if there is evidence of a STEMI, or a cardiac cause is likely, since 30-day survival is highest for patients who are taken directly for pPCI. Ambulance services should continue to work with regional pPCI-capable centres to ensure that suitable patients are accepted to maximise potential for survival.

## Introduction

UK ambulance services attend roughly 60,000 out-of-hospital-cardiac-arrest (OHCA) cases every year, with resuscitation attempted in approximately 50%. In the UK, return of spontaneous circulation (ROSC) and survival to discharge rates have ranged, respectively, from 10–25% and 1–8% between regions (Perkins & Cooke, 2012). Improving outcomes from cardiac arrest has been a priority both nationally and internationally for a number of years, with the European Resuscitation Council (ERC) first developing the chain of survival in 2005 (Deakin et al., 2018).

The post-resuscitation care phase of an OHCA (the final link in the chain of survival) is potentially the most complex, with numerous elements involved, including targeted temperature management, seizure control, airway management and the support of unstable haemo-dynamics (Jentzer et al., 2017).

In 2015, when the ERC updated the resuscitation guidelines, increased emphasis was placed on the need for urgent angiography and revascularisation (Nolan et al., 2015). North American guidelines from Canada and the USA have similarly emphasised the need for prompt angiography and pPCI in eligible patients (Fisher et al., 2018; Wong et al., 2017)

Yorkshire Ambulance Service (YAS) currently recommends that all patients who are resuscitated from OHCA have a 12-lead ECG and are referred directly to pPCI if STEMI is present. There is, however, no pathway for non-STEMI patients where a cardiac cause is suspected, or for STEMI patients whose referral directly into catheter labs is refused.

This service evaluation aimed to determine the factors that affect the transport destination, hospital characteristics and 30-day survival rates of post-ROSC patients with presumed cardiac aetiology.

## Methods

### Setting

YAS provides 24-hour emergency and healthcare services for approximately five million people, within the county of Yorkshire in northern England. It employs over 3000 front line ambulance staff, and in 2018 it responded to around 3000 cardiac arrests where resuscitation was attempted.

### Participants

All adult (18 years and over) patients who achieved ROSC following OHCA and were transported to hospital by YAS between 1 January and 1 July 2017 were eligible for inclusion if:

- ROSC was achieved on scene
- The patient was treated and transported by YAS staff

Patients were excluded if:

- The cause of the arrest was due to trauma
- They had already been referred and accepted by a pPCI unit *prior* to cardiac arrest
- The patient was not transported to hospital

### Data collection

Patient care records previously identified as part of the AIRWAYS-2 trial were reviewed for eligibility for the evaluation by a trust research paramedic.

The patient’s age, gender and presenting rhythm, provision of bystander CPR, whether the OHCA was witnessed and the hospital outcome – comprised of ROSC on arrival at hospital, and 30-day survival – were recorded. In addition, patient records were screened to identify whether a 12-lead ECG was recorded and whether the diagnosis of STEMI and referral made to the receiving hospital were documented. Finally, the destination hospital was identified to determine whether it had a pPCI unit.

Descriptive statistics were utilised to establish current practice and detect any correlations between patient characteristics, type of receiving unit and survival outcomes.

## Results

478 patients met the inclusion criteria for the evaluation and were included in the analysis. Patients were of a similar age in all three groups, but there was a lower proportion of female patients (15%) and a higher proportion of patients with a cardiac history (49%) in the pPCI group (Table 1). In addition, a lower proportion of patients in the pPCI group received bystander CPR, despite this group having the highest proportion of witnessed OHCA.

**Table 1.** Summary table of OHCA cardiac arrest obtaining ROSC, stratified by destination.

	ED at non-pPCI hospital	ED at pPCI hospital	pPCI	Overall
N	316	123	39	478
Age (mean (SD))	68.1 (16.7)	64.3 (16.1)	63.8 (13.2)	66.8 (16.4)
Female (%)	129 (40.8)	34 (27.6)	6 (15.4)	169 (35.4)
Cardiac history (%)	109 (34.5)	36 (29.3)	19 (48.7)	164 (34.3)
Initial rhythm (%)				
Asystole	117 (37.0)	36 (29.3)	2 (5.1)	155 (32.4)
PEA	92 (29.1)	26 (21.1)	1 (2.6)	119 (24.9)
VF	106 (33.5)	61 (49.6)	35 (89.7)	202 (42.3)
VT	1 (0.3)	0 (0.0)	1 (2.6)	2 (0.4)
Witnessed (%)	230 (72.8)	93 (75.6)	37 (94.9)	360 (75.3)
Bystander CPR (%)	196 (62.0)	72 (58.5)	14 (35.9)	282 (59.0)
12-lead ECG (%)	227 (71.8)	95 (77.2)	39 (100.0)	361 (75.5)
STEMI (%)	66 (20.9)	42 (34.1)	39 (100.0)	147 (30.8)
pPCI referral (%)	29 (9.2)	26 (21.1)	39 (100.0)	94 (19.7)
pPCI accepted (%)	0 (0.0)	1 (0.8)	39 (100.0)	40 (8.4)
Airway (%)				
Advanced	218 (69.0)	80 (65.0)	10 (25.6)	308 (64.4)
Basic	61 (19.3)	25 (20.3)	6 (15.4)	92 (19.2)
Patent	14 (4.4)	12 (9.8)	21 (53.8)	47 (9.8)
Unknown	23 (7.3)	6 (4.9)	2 (5.1)	31 (6.5)
ROSC on hospital arrival (%)	266 (84.2)	106 (86.2)	39 (100.0)	411 (86.0)
Survived to 30 days (%)				
No	215 (68.0)	66 (53.7)	7 (17.9)	288 (60.3)
Unknown	50 (15.8)	23 (18.7)	11 (28.2)	84 (17.6)
Yes	51 (16.1)	34 (27.6)	21 (53.8)	106 (22.2)

**Figure 1:**
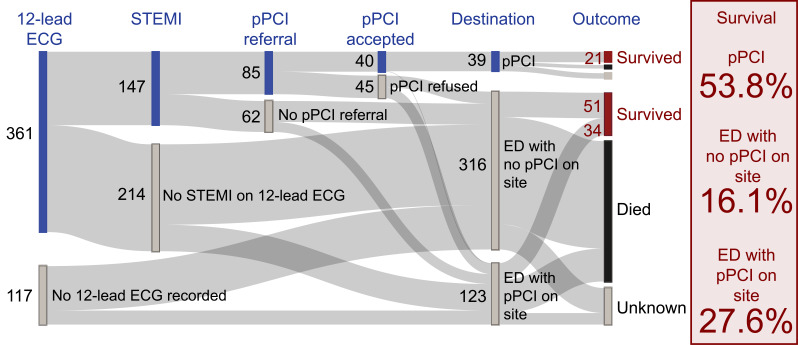
Figure 1. Sankey diagram showing patient journey and survival outcome.

In the subgroup of patients who had an initial rhythm of VF or VT (i.e. a shockable rhythm), the highest proportion of patients to survive to 30 days was in the pPCI group (see Table 2), although the survival outcomes were not known in 42/204 (20.6%) cases. 117 patients were diagnosed with STEMI, of which 85/117 (72.6%) had a pPCI referral made, with 40/85 (47.1%) patients being accepted (Figure 1).

**Table 2.** Summary table of OHCA cardiac arrest outcomes for patients with a shockable rhythm, stratified by destination.

	ED at non-pPCI hospital	ED at pPCI hospital	pPCI	Overall
N	107	61	36	204
Survived to 30 days (%)				
No	50 (46.7)	18 (29.5)	7 (19.4)	75 (36.8)
Unknown	19 (17.8)	14 (23.0)	9 (25.0)	42 (20.6)
Yes	38 (35.5)	29 (47.5)	20 (55.6)	87 (42.6)

## Discussion

The most striking findings of the study were around the referral and acceptance of patients with documented evidence of STEMI, and also the difference in 30-day survival between the types of hospital destination. 12-lead ECG was performed on 76% of cases, revealing 149 patients with documented evidence of STEMI. Of this group, only 88 patients were referred for pPCI and less than half of these referrals were accepted, meaning only 27% of patients with documented evidence of STEMI were taken directly for pPCI. Survival rates were highest for patients transported directly for pPCI. Patients taken to ED at a pPCI-capable hospital had higher 30-day survival rates than those taken to ED at a non-pPCI-capable hospital.

The results are interesting when considered alongside existing literature. Firstly, there was a gender disparity, with 309 males 169 females identified. This could be explained by the fact that the incidence of heart disease in younger males is higher; while women also suffer heart disease, it tends to present later in life with worse outcomes (Khamis et al., 2016). Another notable finding was the higher 30-day survival rates in the shockable rhythm group. Hassager et al. (2018) noted that shockable cardiac arrests are more likely to be associated with a positive outcome, which again is reflected in this sample.

The number of patients receiving a 12-lead ECG was not as high as it could be, at 76%. When this investigation was carried out and STEMI identified, only 59% were then referred directly for pPCI, and the acceptance rate of those referred was just 45%. This meant that of those patients where STEMI had been identified, only 27% went directly to pPCI. This is despite best practice guidelines at a service level, nationally emphasising the importance of recording a 12-lead ECG and referring patients to specialist pPCI units were possible (JRCALC, 2019; Resuscitation Council (UK), 2015). It is unclear from the study why these rates were so low; however, there are likely to be many potential factors, and further research to identify barriers to referral and acceptance would be useful.

Thirty-day survival for the whole cohort was 22.2%. This was much higher for the group taken directly for pPCI, at 53.8%, but even higher for those taken to ED at a pPCI-capable hospital, at 27.6%. The group of patients taken to ED at a non-pPCI-capable hospital had the lowest 30-day survival rates, at 16.1%, lower than the mean survival rate for the whole cohort. This would appear to reflect recent literature demonstrating reduced mortality in groups of OHCA patients taken to pPCI-capable centres (Arabi et al., 2018; Choudry et al., 2015; Dicker et al., 2019; Geri et al., 2015; Tranberg et al., 2017)

## Limitations

This was a small-scale observational study. While patterns were observed, there was no attempt to draw any statistical significance. It was limited to Yorkshire; although there were well-documented themes identified, generalisability of the findings to other regions may not be possible. Survival data was only available for 82% of the sample, potentially affecting validity.

## Conclusions

This study highlights a poor rate of both crew referral and unit acceptance for post-ROSC STEMI patients where pPCI could be indicated during the 6-month period. Thirty-day survival was also observed to be greater for those patients directly taken to pPCI-capable hospitals. Work should continue with ambulance staff and hospital networks to improve the conveyance rates of suitable post-ROSC patients to pPCI-capable hospitals, in order to maximise patients’ potential for survival.

## Guarantor statement

AP acts as the guarantor for this article.

## Conflict of interest

None declared.

## Ethics

Not required.

## Funding

None.
